# Effect of antibiotic prophylaxis in the prognosis of Post-neurosurgical meningitis patients

**DOI:** 10.1186/s40001-023-01399-7

**Published:** 2023-10-04

**Authors:** Guanghui Zheng, Yijun Shi, Jialu Sun, Siqi Wang, Xiang Li, Hong Lv, Guojun Zhang

**Affiliations:** 1 Clinical Diagnosis Laboratory of Beijing Tiantan Hospital, Capital Medical University, NO. 119 Nansihuan West Road, Fengtai District, Beijing, 100076 China; 2 NMPA Key Laboratory for Quality Control of In Vitro Diagnostics, Beijing, 100076 China; 3 Beijing Engineering Research Center of Immunological Reagents Clinical Research, Beijing, 100076 China; 4 Clinical Diagnosis College of Capital Medical University, Beijing, 100076 China

**Keywords:** Antibiotic prophylaxis, Post-neurosurgical meningitis, Survival analysis, Risk factor

## Abstract

**Aims:**

To evaluate the effect of antibiotic prophylaxis(AP) in the prognosis of Post-neurosurgical meningitis(PNM) patients.

**Methods:**

A cohort analysis was performed using the clinical database in Beijing Tiantan Hospital and Capital Medical University. Data were collected on patients with the diagnosis of PNM (*n* = 3931) during 2012.01 to 2022.04. The microbial distribution, types of AP, and 42 and 90 days survival analysis of AP patients were evaluated using probable statistical methods. Independent risk factors for mortality were established by constructing a logistic regression analysis.

**Result:**

A total of 1,190 patients were included in this study, *Klebsiella pneumoniae*, *Acinetobacter baumannii*, and *Staphylococcus aureus* occupied the highest proportion. Of them, 929 cases received AP, cefuroxime and ceftriaxone are the most frequent used antibiotics. In addition, We found that PNM patients without AP significantly increased the 42 days and 90 days all-cause mortality rates. The use of different levels of AP did not improve patient outcomes, and ICU admission and assisted mechanical ventilation (AMV) were identified as independent mortality risk factors for PNM patient received AP.

**Conclusions:**

AP plays an important role in the prognosis of PNM patients and has a significant function in improving prognosis. The prevention of PNM with antibiotics prior to neurosurgery should be emphasized in clinical practice, and appropriate selection of antibiotics is necessary to prevent the occurrence of infection and inhibit the emergence of antibiotic-resistant bacteria.

## Introduction

Post-neurosurgical meningitis (PNM) is a type of infection that commonly occurs in previously damaged or malformed areas of the brain, typically caused by bacterial contamination during or after neurosurgical procedures [1]. Treatment of PNM generally involves the use of antibiotics; however, the condition can be life-threatening and has a high mortality rate, with up to 30% of patients dying even with antibiotic treatment [2]. Therefore, effective prevention of PNM is crucial to reduce morbidity and mortality [3].

Antibiotic prophylaxis (AP) is a preventive measure used to decrease the incidence of bacterial infections after surgery, and it is an essential component of standard care in patients undergoing neurosurgical procedures [4]. AP has been shown to reduce the incidence of PNM to 10–20% [5–7]. However, despite its widespread use, many challenges still exist in the application of AP, including the high rates of PNM, which can be as high as 3–15% even with the administration of antibiotics [8, 9]. Furthermore, it is unclear if the use of 3rd, 4th generation cephalosporins or carbapenem antibiotics can improve the survival rates of PNM patients, which highlights the need for further investigation.

In this study, we conducted a clinical cohort involving more than 1000 PNM patients, aiming to evaluate the role of AP in the prognosis of PNM patients and to identify any associated risk factors. To the best of our knowledge, this is the first global analysis specifically investigating the impact of AP on the prognosis of PNM patients.

## Methods

### Study design and participants

This analytical descriptive cross-sectional study was carried out at Beijing Tiantan Hospital & Capital Medical University, which served as the largest neurosurgical center in northern China, between January 2012 and April 2022. All neurosurgical patients aged 18 years or older with at least one positive cerebrospinal fluid (CSF) culture were enrolled in the study after approval from the ethics committees of both institutions (KY-2021-079-02) with a waiver of informed consent.

### Inclusion and exclusion criteria

Patients diagnosed with PNM were identified based on the classic diagnostic criteria recommended by the U.S. Centers for Disease Control and Prevention (CDC) and the Infectious Diseases Society of America (IDSA). The patients were required to have bacterial proliferation in their CSF and at least one sign of meningeal irritation, such as a headache, neck stiffness, or cranial nerve involvement. In addition, the patients had to exhibit at least one of the following features: raised protein and/or lowered glucose in their CSF, increased neutrophil count, positive Gram stain CSF culture, positive blood culture, positive antigen test in blood or CSF, or increased antibody titer against the pathogen.

The exclusion criteria used in this study involved: (1) neurosurgical patients with either brain abscesses or peritoneal shunt infections, (2) those with incomplete demographic or clinical information, (3) patients aged below 18 years, and (4) those who had died within 72 h of neurosurgery, as well as those with positive CSF cultures for coagulase-negative *staphylococci* (CoNS), Bacillus, and *Propionibacterium*. Additionally, patients were only included if they were admitted to the hospital 48 h or more after the onset of their illness and underwent a neurosurgical procedure.

The AP patients were required to meet two main criteria: firstly, they had to show no signs of infection or meningitis from the time of admission until the neurosurgery. Secondly, they had to receive antibiotics 0–4 h prior to the neurosurgical operation. Patients who met both criteria were classified as AP patients, while those that did not meet or only met one criterion were categorized as non-AP patients.

### Procedures

The present study relied on data extracted from the PNM database, which includes over 6,000 cases of neurosurgical patients with positive CSF cultures. 63 parameters associated with each patient, including demographic data (such as name, age, and gender), medical history (including the presence of a tumor, length of hospitalization, etc.), and details of the neurosurgical operation (such as Craniotomy, Transsphenoidal approach, and antibiotic prophylaxis) were embedded in the database.

Originally designed for monitoring hospital-acquired infections and preventing PNM the database has been utilized by various researchers in a multitude of studies [10, 11]. These have included investigations into risk factors associated with PNM, as well as survival analysis of PNM resulting from multi-drug resistant organisms (MDROs) or *Staphylococcus aureus* infections. In this study, all patients that met the inclusion and exclusion criteria were selected from the database.

For this analysis, we focused on the most extreme vital signs recorded within 24 h of the first positive CSF culture, including the highest body temperature. We also examined all microbiological information pertaining to the first positive CSF culture, including the type of microorganism present in the AP and non-AP groups. In this study, we classified 3rd or 4th generation cephalosporins and carbapenems into one group, and named to high grade antibiotics, which means that these antibiotics have strong bactericidal abilities. Other antibiotics, such as 1st, 2nd generation cephalosporins, Penicillin, Erythromycin were classified into low grade antibiotics, which means these antibiotics have slighter bactericidal abilities. All antibiotics administered to AP patients were evaluated.

The entire treatment plan consisted of two types of antibiotic usage, which were antibiotic empirical therapy (AET) and antibiotic definitive therapy (ADT). The two categories were described as follows: (1) PNM patients who underwent AET were administered antibiotics prior to obtaining the antimicrobial susceptibility testing result; (2) patients who underwent ADT were given antibiotic treatment based on antimicrobial susceptibility testing guidance.

### Statistical analysis

The aim of this study was to investigate the impact of AP on PNM patients’ 42 days and 90 days mortality, as mortality beyond 90 days was deemed less likely to be related to PNM. As secondary outcomes, we evaluated the management of risk factors in patients with AP. Furthermore, all microbiological and clinical epidemic parameters were presented in this study.

Descriptive statistics were conducted using appropriate statistical tests such as the chi-squared or Fisher’s exact tests, t-tests, or Mann–Whitney *U* tests. To assess the relationship between AP and 42 days or 90 days all-cause mortality, we developed a logistic regression model where AP was treated as the dependent variable, and all potential predictors of infectious disease consultation or mortality were considered as independent variables.

In the second stage, we performed a Kaplan–Meier analysis to ascertain the effect of AP on 42 days and 90 days all-cause mortality. In this analysis, AP was treated as a time-dependent variable to adjust for the variability in the timing of consultation.

## Result

During the course of the study, a total of 85,639 patients were admitted to the neurosurgery ward and underwent at least one neurosurgical procedure. Among these patients, 3,931 individuals displayed positive CSF cultures. Of the positive cases, 1, 970 patients were identified as being CoNS culture positive, 215 were Micrococcus positive, 145 were Bacillus positive, and 65 were positive for *Propionibacterium acnes*. A total of 347 cases were excluded from further analysis, 239 cases for having only a CSF ventriculo-peritoneal shunt, 41 for discharge within 24 h, and 67 for having incomplete clinical records. This left 1,190 patients for inclusion in the final analysis.

### Microbiology

Table 1 presents a summary of the distribution of the 1,190 microorganisms that were isolated from the CSF of patients with PNM. Among the identified isolates, *Klebsiella pneumoniae* (12.4%), *Acinetobacter baumannii* (11.6%), and *Staphylococcus aureus* (11.3%) were the most frequently isolated microorganisms. Significant differences (*P* < 0.05) were observed in the distribution of *S. aureus*, *Enterococcus faecalis*, and *Enterococcus faecium* between the groups with and without AP.

**Table 1:** Distribution of the microorganisms caused PNM

Microorganism	AP (929)	Non-AP (261)	Total (1190)	P
*Klebsiella pneumoniae*	119 (12.8%)	28 (10.7%)	147 (12.4%)	0.395
*Acinetobacter baumannii*	110 (11.8%)	28 (10.7%)	138 (11.6%)	0.663
*Staphylococcus aureus*	69 (7.4%)	65 (24.9%)	134 (11.3%)	< 0.001
*Enterococcus faecalis*	81 (8.7%)	10 (3.8%)	91 (7.6%)	0.008
*Enterococcus faecium*	80 (8.6%)	10 (3.8%)	90 (7.6%)	0.008
*Streptococcus viridans*	52 (5.6%)	9 (3.4%)	61 (5.1%)	0.204
*Escherichia coli*	38 (4.1%)	6 (2.3%)	44 (3.7%)	0.198
*Klebsiella aerogenes*	31 (3.3%)	7 (2.7%)	38 (3.2%)	0.694
*Pseudomonas aeruginosa*	29 (3.1%)	7 (2.7%)	36 (3.0%)	0.84
*Enterobacter cloacae*	21 (2.3%)	5 (1.9%)	26 (2.2%)	0.999
*Serratia marcescens*	18 (1.9%)	3 (1.1%)	21 (1.8%)	0.594
*Acinetobacter lwoffii*	18 (1.9%)	2 (0.8%)	20 (1.7%)	0.277
Others	263 (28.3%)	81 (31.0%)	344 (28.9%)	0.396
Total	929 (100%)	261 (100%)	1190 (100%)	–

Of the AP PNM episodes, 98.0% (910/929) of patients received single antibiotics, with the highest percentage of low grade antibiotics at 63.0% (585/929) and cefuroxime at 61.7% (573/929). Among the high grade AP categories, ceftriaxone had the highest ratio, approximately 25.4% (236/929), and other antibiotics were applied in lower proportions, as shown in Table 2.

**Table 2:** Distribution of AP in PNM patients

Groups	Antibiotics	Numbers	Proportion (%)
High grade antibiotics	Ceftazidime	50	5.4
	Meropenem	37	4.0
	Ceftriaxone	236	25.4
	Cefoperazone/Sulbactam	2	0.2
Low grade antibiotics	Erythromycin	7	0.8
	Piperacillin	5	0.5
	Cefuroxime	573	61.7
	Penicillin	5	0.5
	Moxalactam	3	0.3
Others		23	2.5

### Characteristics of PNM patients

The clinical characteristics of the 1,190 patients with PNM are summarized in Table 2. The median age was 42 (27, 54), and 679 (57.0%) were men. Of the PNM patients, 78.1% (929) had experienced AP, and 261 had not. The distribution of age, gender, and comorbidities was similar between groups, and all clinical laboratory tests showed no significant difference. In terms of clinical characteristics, patients who experienced traumatic brain injury, longer operation duration, reoperation, craniotomy, and coma were evenly distributed between patients with and without AP. There was no difference observed between the two groups in other items (Table 3).

**Table 3:** Clinical characteristics of PNM patients received AP or not

Characteristics	AP (929)	Non-AP (261)	Total (1190)	P
Age	41 (27, 54)	42 (27,54)	42 (27, 54)	0.846
Gender	524 (56.4%)	154 (59.0%)	679 (57.0%)	0.48
Diabetes	44 (4.7%)	15 (5.7%)	59 (5.0%)	0.519
Hypertension	146 (15.7%)	49 (18.8%)	195 (16.4%)	0.256
Tumour	663 (71.4%)	183 (70.1%)	846 (71.1%)	0.7
Glioma	230 (24.8%)	62 (23.8%)	292 (24.5%)	
Meningioma	138 (14.9%)	56 (21.5%)	194 (16.3%)	
Pituitary tumor/craniopharyngioma	158 (17.0%)	31 (11.9%)	189 (15.9%)	
Others	137 (14.7%)	34 (13.0%)	171 (14.4%)	
Malignant tumor	290 (31.2%)	84 (32.2%)	374 (31.4%)	0.763
Traumatic brain injury	36 (3.9%)	21 (8.0%)	57 (4.8%)	0.008
Operation duration	4.2 (3.0, 6.0)	3.5 (2.0, 6.0)	4.0 (2.8, 6.0)	0.001
Reoperation	186 (20.0%)	69 (26.4%)	255 (21.4%)	0.013
Craniotomy	664 (71.5%)	161 (61.7%)	825 (69.3%)	0.003
Type I incision	478 (51.5%)	124 (47.5%)	602 (50.6%)	0.263
ICU admission	378 (40.7%)	111 (42.5%)	489 (41.1%)	0.618
CSF Leakage	129 (13.9%)	44 (16.9%)	173 (14.5%)	0.234
EVD(external ventricular drainage)	311 (33.5%)	103 (39.5%)	414 (34.8%)	0.078
LD(lumbar drainage)	250 (26.9%)	79 (30.3%)	329 (27.6%)	0.309
AMV(assisted mechanical ventilator)	384 (41.3%)	102 (39.1%)	486 (40.8%)	0.522
Body temperature	37.7 ± 0.9	37.6 ± 0.9	37.7 ± 0.9	0.807
Coma	49 (5.3%)	57 (21.8%)	106 (8.9%)	< 0.001
Length of hospitalize	22.0 (16.0, 34.0)	23.0 (16.0, 32.0)	22.0 (16.0. 34.0)	0.576
Cure time	5.0 (2.0, 11.0)	5.0 (3.0, 12.0)	5.0 (2.0, 11.0)	0.651
Postoperative infection time	7.0 (4.0, 12.0)	7.0 (4.0, 13.0)	7.0 (4.0, 12.0)	0.983
Fee	71010.0 (50315.8, 119021.2)	79160.5 (50704.7, 142460.0)	72259.0 (50413.0, 123340.5)	0.117
All-cause mortality	110 (11.8%)	61 (23.4%)	171 (14.4%)	< 0.001

Data are presented as means and standard deviations or median (Q1, Q3) for continuous variables and as frequencies and percentages for categorical variables

### High grade and low grade AP in PNM patients

Out of the 929 AP patients, a total of 331 patients received high grade antibiotics, and 587 patients received low grade antibiotics. There were almost no significant differences between the two groups in terms of clinical operations and disease classification, except for some differences observed among patients who underwent postoperative EVD, where more patients received low grade antibiotics(Table 4).

**Table 4:** Clinical characteristics of PNM patients received high or low grade AP

Characteristics	High grade antibiotics(331)	Low grade antibiotics(587)	P
Age	41 (26, 53)	42 (27,55)	0.678
Gender	200 (60.4%)	324 (55.2%)	0.127
Diabetes	17 (5.1%)	27 (4.6%)	0.749
Hypertension	55 (16.6%)	91 (15.5%)	0.925
Tumour	228 (62.8%)	435 (77.5%)	0.092
Glioma	68 (20.5%)	162 (27.6%)	
Meningioma	35 (10.6%)	83 (14.1%)	
Pituitary tumor/craniopharyngioma	48 (14.5%)	110 (18.7%)	
Others	77 (23.3%)	80 (13.6%)	
Malignant tumor	95 (27.2%)	195 (34.1%)	0.161
Traumatic brain injury	19 (5.7%)	17 (2.9%)	0.05
Operation duration	4.0 (2.5, 5.5)	4.5 (3.0,6.0)	0.441
Reoperation	71 (21.5%)	115 (19.6%)	0.496
Craniotomy	236 (71.3%)	428 (72.9%)	0.645
Type I incision	179 (54.1%)	299 (50.9%)	0.372
ICU admission	132 (39.9%)	246 (41.9%)	0.577
CSF Leakage	45 (13.6%)	84 (14.3%)	0.843
EVD(external ventricular drainage)	33 (10.0%)	179 (30.5%)	< 0.001
LD(lumbar drainage)	91 (27.5%)	159 (27.1%)	0.817
AMV(assisted mechanical ventilator)	130 (39.3%)	254 (43.3%)	0.265
Body temperature	37.7 ± 0.9	37.7 ± 0.9	0.845
Coma	14 (4.2%)	35 (6.0%)	0.288
Length of hospitalize	23.0 (17.0, 36.0)	21.0 (16.0,33.0)	0.229
Cure time	6.0 (3.0, 11.0)	5.0 (2.0, 10.0)	0.246
Postoperative infection time	8.0 (5.0,13.0)	7.0 (4.0, 12.0)	0.115
Fee	68992.0 (48682.0, 118003.0)	72687.0 (50833.0, 119312.0)	0.953
All-cause mortality	33 (10.0%)	77 (13.1%)	0.185

Data are presented as means and standard deviations or median(Q1, Q3) for continuous variables and as frequencies and percentages for categorical variables

### Survival analysis

**Fig. 1:**
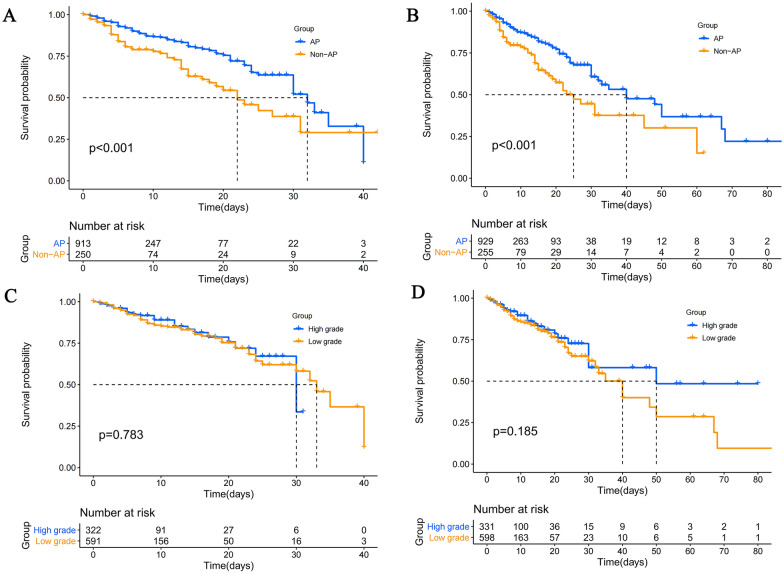
Kaplan–Meier analysis of PNM patients. **A**: 42 days analysis of PNM patients received AP. **B**: 90 days survival analysis of PNM patients received AP. **C**: 42 days survival analysis of PNM patients received high grade AP. **D**: 90 days survival analysis of PNM patients received high grade AP

Survival analysis of PNM patients at 42 and 90 days mortality revealed a significant difference between patients who received AP and those who did not. However, there was no significant difference found between the two groups who received high-grade antibiotics for prophylaxis, as depicted in Fig. 1.

### Mortality risk factors

In univariate analysis, ten characteristics including traumatic brain injury, hypertension, operation duration, reoperation, ICU admission, LD, EVD, AMV, Body temperature, and coma were found to be statistically significantly different between the two groups. In multivariate regression analysis, ICU admission (OR 14.337, 95% CI 1.716–119.798, *P* = 0.014) and AMV (OR 2.841, 95% CI 1.078–7.492, *P* = 0.035) were identified as independent mortality risk factors (Table 5).

**Table 5:** Univariate and multvariate analysis of clinical characteristics of AP patients

Characteristics	Univariate analysis			Multivariate regression analysis		
	Survivors (819)	Non-survivors (110)	P	OR	95%C.I	P
Age	41 (27, 54)	46 (28, 58)				
Gender	454 (55.4%)	72 (65.5%)				
Tumor	581 (70.9%)	82 (74.5%)	0.443			
Malignant tumor	250 (30.5%)	40 (36.4%)	0.200			
Traumatic brain injury	26 (3.2%)	10 (9.1%)	< 0.001	2.508	0.563–11.169	0.228
Diabetes	36 (4.4%)	8 (7.3%)	0.175			
Hypertension	115 (14.0%)	31 (28.2%)	< 0.001	1.329	0.509–3.471	0.561
Operation duration	4.0 (3.0, 6.0)	5.0 (3.4, 7.5)	0.008	1.120	0.963–1.304	0.142
Reoperation	143 (17.5%)	43 (39.1%)	< 0.001	1.516	0.626–3.668	0.356
Craniotomy	581 (70.9%)	83 (75.5%)	0.212			
Type I incision	421 (51.4%)	57 (51.8%)	0.970			
ICU admission	293 (35.8%)	85 (77.3%)	< 0.001	14.337	1.716–119.798	0.014
CSF leakage	108 (13.2%)	21 (19.1%)	0.095			
EVD( external ventricular drainage)	257 (31.4%)	54 (49.1%)	< 0.001	1.093	0.452–2.643	0.843
LD(lumbar drainage)	203 (24.8%)	47 (42.7%)	< 0.001	0.875	0.0369–2.076	0.763
AMV(assisted mechanical ventilator)	299 (36.5%)	85 (77.3%)	< 0.001	2.841	1.078–7.492	0.035
Body temperature	37.6 ± 0.9	37.8 ± 1.0	0.017	1.287	0.599–1.323	0.565
Coma	34 (4.2%)	37 (33.6%)	< 0.001	2.306	0.959–5.544	0.062
Length of hospitalize	21 (16, 32)	31 (18, 46)	0.840			
Cure time	5 (2, 10)	7 (3, 17)	< 0.001	1.004	0.973–1.037	0.792

Data are presented as means and standard deviations or median(Q1, Q3) for continuous variables and as frequencies and percentages for categorical variables

### Therapy

In both the AP and Non-AP groups, a majority of patients with PNM received AET, with 851 (91.6%) and 231 (88.5%) patients, respectively. The most commonly administered antibiotic combination was Meropenem + Vancomycin, accounting for 45.7% in the AP group and 39.0% in the Non-AP group. Additionally, 776 (83.5%) and 213 (81.6%) PNM patients received ADT. Interestingly, our study found no significant difference between AET and ADT in terms of treatment outcomes. However, the use of Ceftazidime showed a statistically significant difference in ADT(Table 6).

**Table 6:** Therapy of the PNM, including AET and ADT

Therapy	AP(929)	Non-AP(261)	Total(1190)	P
AET	851 (91.6%)	231 (88.5%)	1082 (90.9%)	0.143
Meropenem	53 (6.2%)	12 (5.2%)	65 (6.0%)	0.641
Ceftazidime	63 (7.4%)	26 (11.3%)	89 (8.2%)	0.078
Cefotaxime	18 (2.1%)	6 (2.6%)	24 (2.2%)	0.619
Ceftriaxone	81 (9.5%)	17 (7.4%)	98 (9.1%)	0.366
Vancomycin	26 (3.1%)	9 (3.9%)	35 (3.2%)	0.530
Meropenem + Vancomycin	389 (45.7%)	90 (39.0%)	479 (44.3%)	0.073
Ceftazidime + Vancomycin	29 (3.4%)	12 (5.2%)	41 (3.8%)	0.242
Ceftriaxone + Vancomycin	18 (2.1%)	8 (3.5%)	26 (2.4%)	0.231
Cefotaxime + Vancomycin	27 (3.2%)	12 (5.2%)	39 (3.6%)	0.162
Sulbactam Sodium + Meropenem + Vancomycin	12 (1.4%)	6 (2.6%)	18 (1.7%)	0.234
Others	135 (15.9%)	33 (14.3%)	168 (15.5%)	0.609
ADT	776 (83.5%)	213 (81.6%)	989 (83.1%)	0.456
Meropenem	57 (7.3%)	15 (7.0%)	72 (7.3%)	0.999
Ceftazidime	23 (3.0%)	15 (7.0%)	38 (3.8%)	0.014
Cefotaxime	11 (1.4%)	4 (1.9%)	15 (1.5%)	0.542
Ceftriaxone	22 (2.8%)	7 (3.3%)	29 (2.9%)	0.654
Vancomycin	30 (3.9%)	8 (3.8%)	38 (3.8%)	0.999
Meropenem + Vancomycin	370 (47.7%)	96 (45.1%)	466 (47.1%)	0.536
Ceftazidime + Vancomycin	12 (1.5%)	6 (2.8%)	18 (1.8%)	0.245
Ceftriaxone + Vancomycin	12 (1.5%)	3 (1.4%)	15 (1.5%)	0.999
Cefotaxime + Vancomycin	14 (1.8%)	5 (2.3%)	19 (1.9%)	0.578
Sulbactam Sodium + Meropenem + Vancomycin	10 (1.3%)	6 (2.8%)	16 (1.6%)	0.128
Others	215 (27.7%)	48 (22.5%)	263 (26.6%)	0.137

## Discussion

Excessive use of antimicrobial agents has become an growing concern in public health [12]. In particular, after various surgical operations, AP plays a crucial role in infection control [13]. However, the overuse of antibiotics can lead to resistance, which hampers the effective treatment of bacterial infections. In this single-center cohort study conducted in China, we evaluated the microbiology and clinical epidemiology associated with PNM and identified risk factors for mortality in patients receiving AP.

This cohort study had a relatively large sample size, enrolling over 1000 cases. We observed that as many as 75% of patients received AP, while the proportion of PNM patients could reach 15% of all neurosurgical patients, irrespective of whether or not they were administered AP. Our study revealed that the use of AP significantly decreased the all-cause mortality of PNM patients at both the 42 days and 90 days follow-up periods. Interestingly, we did not observe improved patient outcomes when different types of antibiotics were used as prophylaxis. These results indicate that AP may not provide the intended benefits for neurosurgical patients with PNM. Additionaly, we identified independent mortality risk factors for PNM patients receiving AP, which included ICU admission and AMV. Increased attention should be paid to risk factors that may lead to mortality. To the best of our knowledge, this is the largest cohort study to explore the potential benefit of AP for PNM patients in the world. Our pragmatic and detailed clinical cohort design facilitated an investigation into the impact of prophylaxis independent of other factors that may lead to mortality. This study provides valuable insights that can inform the optimization of infection control strategies in neurosurgical practice.

PNM is a significant complication of neurosurgery that may have a detrimental effect on patient outcomes [14]. The administration of antibiotics stands as a pivotal measure in mitigating patient mortality. In clinical practice, preventive and therapeutic approaches are available to improve the outcomes of PNM patients [15]. Therapeutic options encompass the utilization of antibiotics, anti-inflammatory steroids, and open wound management, among others [16, 17]. Nonetheless, patients afflicted with PNM may experience significantly longer hospitalization periods and heightened expenses compared to neurosurgery patients without meningitis, and their survival rates may be significantly lower. Therefore, prevention, besides AP, plays a crucial role in the clinical management of PNM.

The selection of antibiotics is primarily contingent upon the type of microorganism responsible for PNM. Our study findings demonstrate that the responsible microorganisms for PNM are common nosocomial pathogens [18]. Nevertheless, the role of CoNS in PNM remains enigmatic. Throughout the study's duration, CoNS constituted over half of the positive pathogen cultures. While prior research has suggested that CoNS can indeed induce PNM [19], particularly in cases involving meningitis related to ventricular peritoneal shunts [20], our own prior investigation revealed that 79% of CoNS isolates were classified as contaminants [21]. Hence, in this present study, CoNS was deliberately excluded from the cohort analysis. In the rest of cases, no significant disparities exist between the top two pathogens among patients who received or did not receive AP. However, there were significant differences in the isolation rates of the top three Gram-positive bacteria, mainly *Staphylococcus aureus*, which had a significantly lower proportion in the AP group than in the non-AP group. Probably reason target to this issue may be the choice of AP. Cephalosporins, such as cefuroxime and ceftriaxone, accounted for 87.1% of the whole antibiotic usage. Both of these antibiotics possess notable efficacy against *Staphylococcus aureus,*except the Methicillin-resistant *Staphylococcus aureus* [22]. However, neither had a high sensitivity rate to *Enterococcus faecalis,* and as we all know, antibiotics combination is the most preferred choice in *Enterococcus* infections [23]. Our findings suggest that AP has a greater impact on patients infected with *Staphylococcus aureus*, and the antibiotics used in AP are more effective against this *Staphylococcus aureus* than against *Enterococcus spp*.

Previous studies have predominantly concentrated on the reduction of infection incidence with AP. Despite guidelines advocating for the administration of antibiotics prior to neurosurgery, their impact on patient prognosis has not been reported [24, 25]. Our study reveals that AP patients exhibit significantly lower all-cause mortality rates at 42 and 90 days compared to Non-AP patients, indicating a positive effect on patient outcomes. While the optimal antimicrobial regimen for preventing PNM during the perioperative period remains uncertain, guidelines propose using a single β-lactam antibiotic for most surgical procedures [4]. Our findings align with this recommendation; however, the guidelines do not stipulate the specific antibiotic type to employ. Antibiotic management proposes utilizing different levels of antibiotics with varying application strategies in clinical practice.

With the escalation of antibiotic categorization, the emergence of antibiotic-resistant bacteria becomes increasingly evident in clinical settings.. We classified patients based on their use of high-level or low-level antibiotics and found no differences in survival indicators such as survival time, treatment costs, and the length of hospital stay. This investigation underscores that the utilization of high-grade antibiotics failed to yield enhanced patient outcomes and, conversely, could heighten the susceptibility to unfavorable reactions due to their robust bactericidal impacts. Literature reports suggest that the use of high-level antibiotics can easily lead to the emergence of drug-resistant bacteria, and vancomycin, in particular, is known to have significant adverse effects [26–28]. Additionally, the two primary antibiotics used in our study, ceftriaxone (a high grade antibiotic) and cefuroxime (a low grade antibiotic), have differing effects on Gram-negative bacteria. Third generation cephalosposin is always effective against Gram-negative bacteria but may induce bacterial resistance (e.g., by producing AmpC) [29], while cefuroxime does not have this effect or possess slighter side effects, and even much cheaper. Therefore, we recommend that cefuroxime should be the preferred antibiotic for prophylaxis as it may offer equal or better benefits to patients.

Mitigating patient mortality and enhancing prognosis in the context of PNM represents a pivotal research objective. In addition to AP, other factors that impact patient outcomes should not be overlooked, such as AMV and EVD. Thorough scrutiny of risk factors across all patients subjected to AP can serve to attenuate patient mortality during the initial phases. In our previous studies, a series of risk factors about to PNM have been evaluated, such as GCS scores [11], EVD [30], AMV[10], craniotomy and malignancy [9] and so on. This study evaluated two mortality risk factors, ICU admission and AMV. Both factors are known to have poor prognoses due to the severity of the underlying disease, making patients more susceptible to infection and leads to poor outcome.

In this investigation, we scrutinized two distinct treatment modalities for patients diagnosed with PNM. The initial approach involved AET, predominantly due to the delay in receiving antibiotic susceptibility test results. Consequently, a broad-spectrum antibiotic regimen was administered to ensure comprehensive coverage and mitigate any potential adverse consequences. Our findings revealed that the most frequently prescribed empirical antibiotic combination was Meropenem + Vancomycin, which may be associated with the severity of PNM and its higher propensity for unfavorable prognosis. The second approach entailed ADT, guided by the findings of antibiotic susceptibility testing. Similar to AET, the Meropenem + Vancomycin combination remained the predominant choice. This preference can be attributed to the widely accepted and efficacious nature of the Meropenem + Vancomycin clinical regimen for combating PNM. Additionally, a slightly lower proportion of patients received ADT compared to AET. This variation could be explained by the longer cultivation period (3–5 days) required for pathogen identification, allowing some PNM patients to recover within the timeframe of AET.

Insufficient investigation has been conducted regarding AP in the context of PNM. Firstly, single-center studies possess inherent limitations, and even with a sample size of over 1000 cases, single-center studies are less reliable than multi-center studies in terms of overall credibility. Secondly, the primary diseases causing PNM were not classified in detail, and only neurosurgical operations were analyzed as a category of disease. Lastly, the antibiotics used for AP at our center were limited, so we were only able to group them according to high level and low level antibiotics, which had certain limitations.

In summary, AP emerges as a pivotal determinant in the prognosis of PNM patients, exhibiting the potential to markedly enhance outcomes. Variations in antibiotic types exhibit negligible influence on patient mortality rates, thereby providing insights for antibiotic selection in clinical practice. Among all PNM patients receiving AP, ICU admission and AMV are independent risk factors for patient death, highlighting the need for vigilance in managing these factors by clinicians.

## Data Availability

The datasets used and/or analyzed during the current study are available from the corresponding author on reasonable request.

## Abbreviations

AP: Antibiotic prophylaxis
PNM: Post-neurosurgical meningitis
CSF: Cerebrospinal fluid
CDC: Centers for Disease Control and Prevention
IDSA: Infectious Diseases Society of America
CoNS: Coagulase-negative *staphylococci*
MDROs: Multi-drug resistant organisms
EVD: External ventricular drainage
LD: Lumbar drainage
AMV: Assisted mechanical ventilator
